# Reply to J Barbaresko et al.

**DOI:** 10.1016/j.advnut.2024.100202

**Published:** 2024-03-28

**Authors:** Marilena Vitale, Rosalba Giacco

**Affiliations:** a Department of Clinical Medicine and Surgery, Diabetes, Nutrition and Metabolism Unit, Federico II University of Naples, Italy; b Institute of Food Sciences, National Research Council, Avellino, Italy

Dear Editor:

We read with great attention the criticisms raised by Barbaresko et al. [1] on our systematic review and meta-analysis [2], and we are glad to have the opportunity to clarify some issues that seem to have been misunderstood. First, they argue that we have put together studies with different designs; actually, among the 25 studies included in our meta-analysis, 3 have a cross-sectional design [[3], [4], [5]] and were included due to the paucity of prospective studies on dyslipidemia (triglycerides and high density lipoprotein-cholesterol). Noteworthy, when recalculated with the exclusion of these 3 studies, risk estimates do not change and remain statistically significant. We therefore believe that the interpretation of the results is soundly based, despite all the limitations that we described in the discussion [2] and Mendoza et al.’s study [6] in the commentary to our meta-analysis.

Concerning the criticism that we failed to present confounders and to adequately assess risk of bias in primary studies, it is worth underlining that to limit the possible influence of confounders on the study outcomes, the data from the primary studies included in the meta-analyses were those that had been adjusted for all possible confounders by the study authors, as we clearly stated in the methods [2]. In addition, unlike prior meta-analyses, we have also taken into account in the summary estimates the confounding role of the diversity of dietary assessment instruments whose heterogeneity is of utmost relevance in the interpretation of the current literature. The relevance of the latter issue is clearly underlined in the commentary on the study [6].

Furthermore, Barbaresko et al. [1] expressed skepticism over the use of the Newcastle Ottawa Scale (NOS) and stated that the criteria for the judgment of single items of NOS were not transparently presented. As far as we know, NOS remains the reference method for nonrandomized studies and has been utilized in as many as 800 studies in the last year alone: should we consider this extensive literature to lack any scientific validity?

A further comment pertains to the relevance of the outcomes studied and the novelty of the study. The study outcomes include the most prevalent chronic conditions affecting a significant proportion of the populations all over the world and are, therefore, relevant for human health. Unlike prior works, this study evaluated the possible differential associations depending on the methodology used to assess the intake of ultraprocessed foods (UPFs) and the amount of total UPF consumption.

Barbaresko et al. [1] outline some inconsistencies between the primary studies included in our article and those present in other meta-analyses on the same topic. The inconsistencies between the studies selected in different meta-analyses on the same topic by various authors are a widely recognized phenomenon that occurs despite the utilization of transparent and objective inclusion criteria: our study is no exception. These apparent discrepancies are due to various reasons. The chronology of the literature search is important; the results of the Nurses’ Health Studies and the Health Professional Follow-up Study [7] were published after the completion of our search. Other reasons for the inconsistencies reside in the differences in the inclusion criteria, the most relevant being that we focus on studies reporting all types of UPF according to the NOVA classification system and not only on specific foods, such as meat or beverages, or dietary patterns [8,9].

Staying on the systematic search process, Barbaresko et al. [1] also adduced a lack of transparency and introduced biases because for 174 studies the full text was not retrieved. In reality, these studies were excluded in the phase of abstract evaluation, based on the inclusion criteria of our meta-analysis.

Various other inconsistencies in the data extraction were found. As for obesity, in the studies included there were cohorts with overweight, but we considered exclusively risk estimates for obesity (i.e., BMI ≥ 30 kg/m^2^). Noteworthy, when recalculated with the inclusion of overweight/obesity, risk estimates do not change and remain statistically significant. We therefore believe that the interpretation of the results is soundly based.

Risk estimates refer to the amount of UPF expressed as grams per day, or percentage of daily energy intake, or proportion relative to total dietary weight: we did not perform a dose–response meta-analysis due to the absence of available data in the literature as clearly stated in the Discussion; therefore, in our opinion, this criticism is more than questionable. In our view, the same could be true for the statement that “the number of participants and cases were not correct for several studies”; indeed, the numbers we carefully verified.

Finally, Barbaresko et al. [1] dispute our application of the NutriGrade scoring system. When possible, we have evaluated each study separately to obtain a more precise quality of evidence for the studies included to not allow the overestimation of some points of the NutriGrade scoring system. In any case, even if the score is assessed for the overall associations, the results do not change (for diabetes, from 6.2 to 6.4, for hypertension, from 5.5 to 5.9, for dyslipidemia, from 5.7 to 5.4, for obesity, is equal to 6).

In conclusion, our meta-analysis includes all the necessary methodological approaches, literature search, and grading of evidence to provide very important information on UPFs and human health, as stated in the accompanying editorial of the Journal.

## Conflict of interest

The authors report no conflicts of interest.
